# Resting-State Functional Magnetic Resonance Imaging of Interhemispheric Functional Connectivity in Experimental Traumatic Brain Injury

**DOI:** 10.1089/neur.2021.0023

**Published:** 2021-11-29

**Authors:** Shiliang Huang, Qiang Shen, Lora Talley Watts, Justin A. Long, Michael O'Boyle, Tony Nguyen, Eric Muir, Timothy Q. Duong

**Affiliations:** ^1^ Research Imaging Institute, UT Health San Antonio, San Antonio, Texas, USA.; ^2^ Department of Radiology, UT Health San Antonio, San Antonio, Texas, USA.; ^3^ Department of Clinical and Applied Science Education, University of the Incarnate Word School of Osteopathic Medicine, San Antonio, Texas, USA.; ^4^ Department of Radiology, Montefiore Medical Center and Albert Einstein College of Medicine, Bronx, New York, New York, USA.; ^5^ Department of Radiology, Stony Brook Medicine, Stony Brook, New York, USA.

**Keywords:** animal studies, behavior, MRI, recovery, traumatic brain injury

## Abstract

Although resting-state functional magnetic resonance imaging (rsfMRI) has the potential to offer insights into changes in functional connectivity networks after traumatic brain injury (TBI), there are few studies that examine the effects of moderate TBI for monitoring functional recovery in experimental TBI, and thus the neural correlates of brain recovery from moderate TBI remain incompletely understood. Non-invasive rsfMRI was used to longitudinally investigate changes in interhemispheric functional connectivity (IFC) after a moderate TBI to the unilateral sensorimotor cortex in rats (*n* = 9) up to 14 days. Independent component analysis of the rsfMRI data was performed. Correlations of rsfMRI sensorimotor networks were made with changes in behavioral scores, lesion volume, and T_2_- and diffusion-weighted images across time. TBI animals showed less localized rsfMRI patterns in the sensorimotor network compared to sham (*n* = 6) and normal (*n* = 5) animals. rsfMRI clusters in the sensorimotor network showed less bilateral symmetry compared to sham and normal animals, indicative of IFC disruption. With time after injury, many of the rsfMRI patterns in the sensorimotor network showed more bilateral symmetry, indicative of IFC recovery. The disrupted IFC in the sensorimotor and subsequent partial recovery showed a positive correlation with changes in behavioral scores. Overall, rsfMRI detected widespread disruption and subsequent recovery of IFC within the sensorimotor networks post-TBI, which correlated with behavioral changes. Therefore, rsfMRI offers the means to probe functional brain reorganization and thus has the potential to serve as an imaging marker to longitudinally stage TBI and monitor for novel treatments.

## Introduction

Traumatic brain injury (TBI)^1^ has been shown to cause structural damage to neural connections using diffusion tensor imaging.^2^ Disruption of these anatomical connections have been associated with cognitive deficits and decreased neuropsychological performance after acquired brain injury.^3^ More recently, resting-state (rsfMRI) functional (fMRI) magnetic resonance imaging (MRI), which measures the temporal correlation of spontaneous low-frequency blood oxygenation level–dependent (BOLD) fMRI signals among different brain regions,^4,5^ has emerged as a useful tool to measure changes in functional (in contrast to anatomical) connectivity of large-scale networks post-TBI.^3^ Past rsfMRI studies of TBI patients have found disruptions in functional connectivity in various functional networks,^6–9^ including the default mode networks (DMNs).^8,10^ Many patients with acquired brain injury also show partial recovery, and there is evidence that such recovery is associated with formation of new neural connections or rearrangement of neural networks in proximal and distant areas to the injured site.^11^ Although rsfMRI use has increased, there are few studies that examine the effects of moderate TBI for monitoring functional recovery in experimental TBI, and thus the neural correlates of brain recovery from moderate TBI remains incompletely understood.

TBI in humans is inherently heterogeneous because of the variability in location and severity of the injury as well as individual responses to injury. Animal TBI models offer the advantage of targeting the injury to a specific brain region with high reproducibility. Severity of injury can be tightly controlled across multiple animals. rsfMRI in animal models is useful because task-evoked fMRI studies of anesthetized animals are generally limited to somatosensory stimulations given that it is challenging to evoke higher-order brain functions in anesthetized animals. There are only a few rsfMRI studies on experimental TBI, to our knowledge.^12–15^ Most of these studies utilized seed-based methods to analyze rsfMRI data. Although it has merit, findings are dependent on the location and size of the seeds. Most of these studies did not investigate acute and subacute phases of TBI. In short, the temporal evolution of functional connectivity from hyperacute and chronic experimental TBI remains understudied.

The goal of this study was to use rsfMRI to longitudinally investigate the impact of acute moderate TBI on functional connectivity disruption and its subsequent recovery within the bilateral sensorimotor network of rats. Comparisons were made with changes in lesion (contusion) volume, transverse relaxation time (T_2_)-, diffusion-weighted MRI, and sensorimotor behavioral scores. Comparisons were also made with sham surgery and control animals. We tested the hypothesis that rsfMRI interhemispheric functional network connectivity (IFC) of the sensorimotor pathways is perturbed, rsfMRI IFC is correlated with sensorimotor functional deficits post-TBI, and that improvements in sensorimotor function are correlated with restoration of rsfMRI IFC. The ultimate goal and motivation of this study is to test whether rsfMRI can be utilized as a tool for the evaluation of TBI recovery and/or provide additional information for multi-parametric predictions of tissue fate post-TBI.

## Methods

### Animal preparations

Animal procedures followed guidelines and regulations consistent with the Guide for the Care and Use of Laboratory Animals, Public Health Service Policy on Humane Care and Use of Laboratory Animals, the Animal Welfare Act, and the Animal Welfare Regulations and were approved by the Institutional Animal Care and Use Committee of the University of Texas Health Science Center San Antonio (protocol no. 13034). The ARRIVE (Animal Research: Reporting of In Vivo Experiments) guidelines were followed in the preparation of this article. Rats were housed 2 to a cage in a Tecniplast caging system (Tecniplast, West Chester, PA) with autoclaved sani-chip bedding, including 12-h light and 12-h dark cycles. Rats had *ad libitum* access to irradiated rodent chow from Harlan Laboratories (Indianapolis, IN) and autoclaved water. Sample sizes were derived from expected variances and differences between groups.^16,17^ Animals arrived at our facility at least 5 days before experimentation.

Three groups of a total of 20 male Sprague-Dawley rats (250–350 g, 3–6 months of age) were studied: 1) control animals (no surgery or impact) only imaged once (*N* = 5); 2) sham animals (cranial window but no impact) imaged longitudinally up to 14 days (*N* = 6); and 3) TBI animals imaged longitudinally up to 14 days (*N* = 9). The sham group included all surgical procedures except the actual physical impact, in which depth of the impactor was dialed back so that the tip would contact the cortical surface. For the TBI group, rats were anesthetized initially with 5% isoflurane mixed with room air and subsequently maintained at 1.2% isoflurane throughout all subsequent surgical and imaging procedures.^17^ Respiratory rate, heart rate, and rectal temperature were monitored continuously. Pinching the paws as well as pulse of measurements were taken to ensure adequate anesthesia. Isoflurane is a commonly used anesthetic because of its ease of use and fast recovery. Disadvantages of isoflurane for fMRI are that it affects the baseline cerebral blood flow and neurovascular coupling, which would confound fMRI quantification.^18^ In addition, isoflurane also suppresses neural activity.^19^

Some studies have reported that rsfMRI responses can be modulated by isoflurane and showing different patterns under difference levels of isoflurane.^20,21^ However, many studies have demonstrated that robust rsfMRI data can be obtained under a relatively low dose (∼1%) of isoflurane in both rodent^22,23^ and non-human primate^20^ models. The animal was secured in a stereotaxic frame, and an incision was made as posterior (at the level of the cerebellum) from the impact site as possible to prevent artifacts during MRI acquisition. The periosteum was also removed over the impact site. A Ø5-mm craniotomy was created over the left forelimb primary sensorimotor cortex (S1FL and M1FL: +0.25-mm anterior and 3.5-mm lateral to bregma), exposing the dura matter. The intact dura matter was impacted using a pneumatic controlled cortical impactor (Precision Systems and Instrumentation, LLC, Fairfax Station, VA) fitted with a Ø3-mm tip (5.0 m/s, 250-μs dwell time, 1-mm depth) to mimic a moderate focal TBI.^16,17^ After the impact, the cranial opening was sealed with bone wax, the scalp sutured closed, and antibiotic ointment was applied. Saline was injected under the skin to facilitate the removal of air pockets between the scalp and skull to minimize artifacts during MRI acquisition. Buprenex (0.05 mg/kg) was given subcutaneously every 12 h for 3 days, as needed, for pain.

MRI was acquired on the day of the TBI or sham procedure (3 h post-TBI, day 0) and again on days 2, 7, and 14 after TBI onset. Behavioral assessments were made 1–3 days before TBI and again 2, 7, and 14 days post-TBI before the MRI experiments in the same animals. Behavioral tests were not performed on the day of TBI induction because of incomplete recovery from anesthesia.

### Magnetic resonance imaging

MRI was performed on a rodent Bruker 7-Tesla (T) Pharmascan Scanner with a 16-cm clear bore diameter under 1.2% isoflurane (Bruker Corporation, Billerica, MA). The animal was secured in a custom-built, MRI-compatible, rat head stereotaxic holder with ear and tooth bars. The animal's rectal temperature was monitored and maintained at 37.0°C ± 0.5°C using a circulating water system. Heart rate and respiration rate were monitored continuously and maintained within normal physiological ranges.

#### Resting-state functional magnetic resonance imaging (10 min)

rsfMRI was performed on a 7T scanner under 1.2% isoflurane, which has been optimized for rsfMRI acquisition. BOLD fMRI data were acquired using gradient echo-planar imaging (EPI) scans with partial Fourier (5/8). Other acquisition parameters were: seven 1.0-mm coronal images, field of view (FOV) = 2.56 × 2.56 cm, matrix 96 × 96 and reconstructed to 128 × 128, single shot, repetition time (TR) = 1 sec, echo time (TE) = 20 ms, and 600 time points.

#### T_2_ map (9.5 min)

T_2_-weighted images were acquired using a fast spin-echo sequence with TR = 3 sec (90-degree flip angle), effective TE = 18, 54, 90, and 126 ms, and four echo train lengths, where the center of the echo train was taken as the effective TE for T_2_ calculation. The other parameters were: seven 1.0-mm coronal images, FOV = 2.56 × 2.56 cm, matrix 96 × 96 and reconstructed to 128 × 128, and eight transients for signal averaging.

#### Diffusion tensor magnetic resonance imaging (3.5 min)

Diffusion MRI was obtained using a single low b-value (10 s/mm^2^, which included a contribution from imaging gradients) in 30 directions with a 1200-s/mm^2^ b_max_ value. EPI scans with partial Fourier (5/8) were also acquired using the following settings: seven 1.0-mm coronal images, FOV = 2.56 × 2.56 cm, matrix 96 × 96 and reconstructed to 128 × 128, single shot, TR = 3 sec, TE = 32 ms, Δ = 14 ms, δ = 5 ms, and two transients for signal averaging.^24–26^

### Image analysis

#### Resting-state functional magnetic resonance imaging analysis

BOLD time-series images were realigned and detrended using Statistical Parametric Mapping (SPM; version 8) and coregistered based on individual T_2_-weighted MRI (MANGO v3.11) and then to a reference template for group analysis. The data were band-pass (0.01–0.10Hz) filtered, and spatial smoothing of twice the voxel size was applied for denoise using SPM. No time-slice correction was used because only seven slices were acquired using single-shot EPI because the expected error was small. The rsfMRI data were analyzed using the resting-state analysis toolbox (MELODIC) in FSL 5.0.8 (FMRIB's Software Library; www.fmrib.ox.ac.uk/fsl).^27^ Independent component analysis (ICA) was used with the number of independent components set to 15, typically used in the literature.^28^ We verified that 15 components were reasonable in yielding reliable clusters that did not appear identical and were consistent across time. Group rsfMRI maps were overlaid on two-dimensional reference templates. Based on the rsfMRI maps, four robust ICA components relevant to the TBI model were identified for subsequent analysis and they were denoted as the: 1) cingulate (Cg); 2) primary forelimb sensorimotor cortex (SM), which included roughly the forelimb primary motor and primary somatosensory cortices on the rsfMRI maps; 3) supplementary somatosensory cortex (SSS); and 4) caudate putamen (CPu) regions.

To quantify the interhemispheric symmetry of the functional connectivity, a Z-score threshold of 3 was set to derive the rsfMRI cluster volume for each component. The volume ratio of ICA clusters of the two hemispheres (interhemispheric symmetry index [SI]) was tabulated. Note that the ratio of the smaller to larger number of the two hemispheres was taken, such that unity indicated interhemispheric symmetry and less than unity indicated asymmetry. The leave-one-out method was used to obtain standard errors of SI in which a data set from one animal data was taken out at a time and ICA analysis was performed N-1 times.

#### T_2_ and apparent diffusion coefficient values of the independent component analysis clusters

T_2_ and apparent diffusion coefficient (ADC) maps were calculated using custom Matlab codes (The MathWorks, Inc., Natick, MA). T_2_-weighted signals sampled at four different TEs were fitted to an exponential decay curve (*S_i_ = M_o_ e^−TE^_i_^/ T2^*) to determine T_2_ pixel by pixel. ADC maps with intensity in units of square millimeters per second were calculated pixel by pixel by using the Stejskal and Tanner equation: ADC = −ln (S_1_ / S_o_)/(b_1_ −b_o_), where b_i_ = γ^2^ G_i_^2^ δ^2^ (Δ – δ/3), ln is the natural logarithm, and S_o_ and S_1_ are the signal intensities obtained with b_o_ and b_1_, respectively. The b-value is proportional to the gradient strength (G), magnetogyric ratio (γ), duration of each gradient pulse (δ), and the time (Δ) between applications of the two gradient pulses.

T_2_ and ADC values were tabulated for the four brain regions based on the Cg, SM, SSS, and CPu rsfMRI maps. Regions of interest (ROIs) were placed in the ipsilesional hemisphere and homologous regions in the contralesional hemisphere. T_2_ and ADC values of ipsilesional ROIs were normalized with respect to those of the contralesional ROIs to simplify the comparisons. To assess whether TBI affects T_2_ or ADC in the contralateral hemisphere, we compared T_2_ and ADC values in the contralesional hemisphere of the TBI animal group with those of normal animals and did not find significant differences. Statistical tests were based on quantitative T_2_ and ADC values, not normalized values.

#### Lesion volumes

Lesion volume was determined based on T_2_ maps. A threshold of twice the standard deviation above the mean was used to segment the abnormal T_2_ pixels to derive the T_2_ lesion volumes at each time point.^16,17^ The threshold method instead of manually drawing ROIs was used to avoid unintentional bias.^24–26^

### Functional assessment

Sensorimotor function was assessed using the foot-fault test as described previously.^17^ The foot-fault test is used to assess locomotor function in rodents with central nervous system dysfunction. This test was chosen based on the area of the brain that was impacted and from our previous studies that demonstrated this as an effective measure of locomotor dysfunction post-TBI.^16,17^ Testing was conducted 1–3 days pre-TBI and again 2, 7, and 14 days post-TBI before performing MRI at these time points. A video camera was used to record locomotion while on an elevated grid floor (size 18 × 11 inches with grid openings of 1.56 × 1.00 inches). The animal was recorded for 5 min or until 50 steps were taken with one (non-affected) forelimb. The number of steps taken and the number of forelimb slips through the grid were tabulated. The percentage of foot faults for each limb was calculated as the number of right or left forelimb foot faults divided by the total number of steps taken.

### Statistical and correlation analysis

rsfMRI SIs of the TBI group at four time points were compared to those of the sham group with mixed-effects model using a commercial statistical tool (SPSS 27; IBM, Armonk, NY), including the subject as a random factor with intercept to account for repeated measurements. To further test the significance between groups at each time point, an unpaired *t*-test was performed and followed by the Bonferroni-Holm correction for multiple comparisons. T_2_ and ADC values of ipsilesional hemisphere at different time points were compared with those of contralesional hemisphere using a paired *t*-test. For behavioral data, an unpaired *t*-test was used to compare differences across time with sham group data. Correlation analysis of the rsfMRI SI with lesion volumes, behavioral scores, quantitative T_2_, and ADC across time was performed using two-way analysis of variance with the Pearson correlation for multiple comparisons. A value of *p* < 0.05 (two tails) was considered to be statistically significant.

## Results

### Control animals

**FIG. 1.**
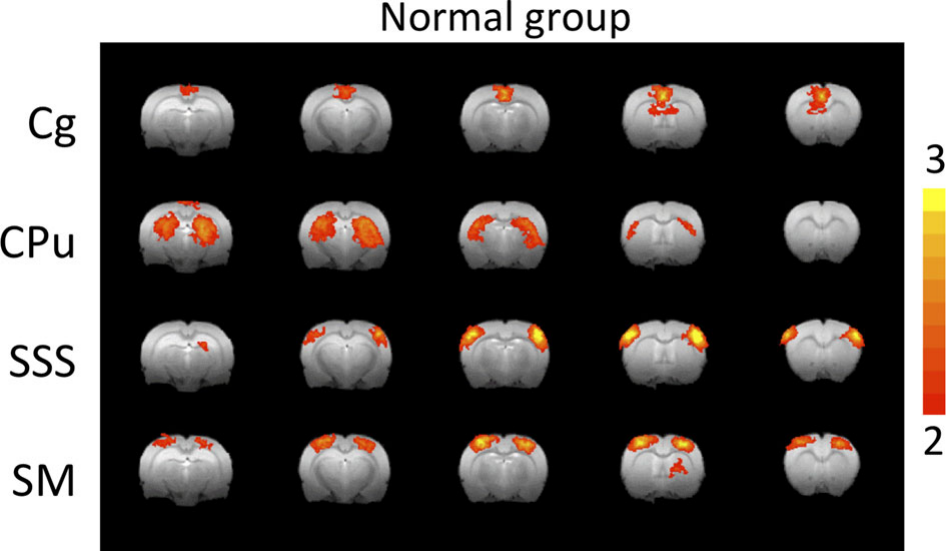
ICA rsfMRI maps of Z-scores from normal rats (*n* = 5) without surgery. Four representative ICA components were robustly detected and they are: cingulate (Cg), caudate putamen (CPu), supplementary somatosensory cortex (SSS), and sensorimotor cortex (SM). ICA, independent component analysis; rsfMRI, resting-state magnetic resonance imaging.

rsfMRI analysis of the control animals (no surgery) showed four consistent rsfMRI components (Fig. 1). The rsfMRI Z-score maps showed highly localized functional neural networks, corresponding to known anatomical structures of the Cg, SSS, primary forelimb SM, and CPu. The Cg showed localized and bilaterally symmetric patterns close to the midline. The CPu, SSS, and SM clusters, known components of the sensorimotor pathway, showed bilateral symmetric patterns. Subsequent analysis focused on evaluating changes of these four ICAs.

### Sham and traumatic brain injury resting-state functional magnetic resonance imaging maps

**FIG. 2.**
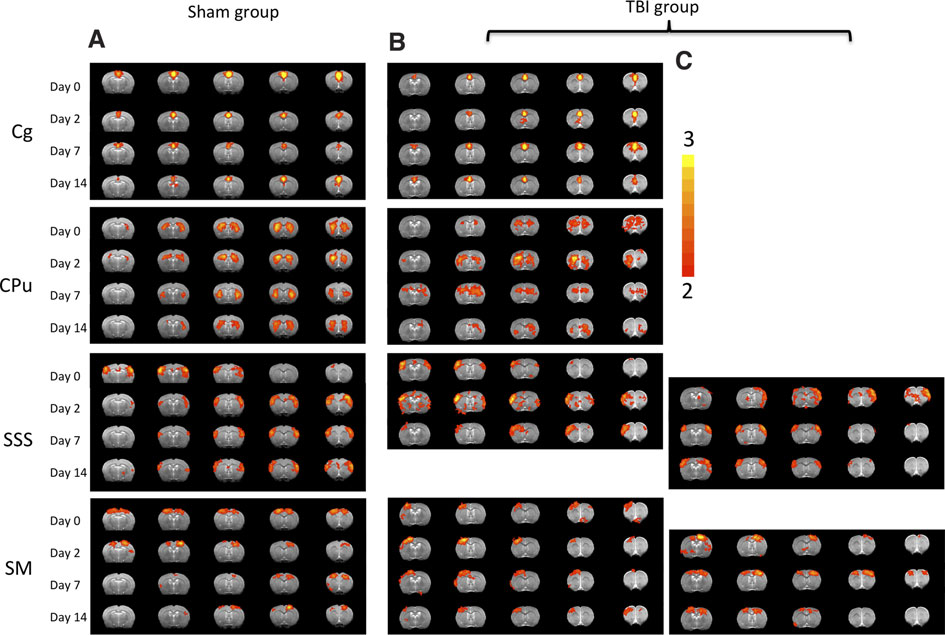
ICA rsfMRI maps of Z-scores from (**A**) sham (*n* = 6) and (**B**) TBI (*n* = 9) rats. Four representative ICA components are shown: cingulate (Cg), caudate putamen (CPu), supplementary somatosensory cortex (SSS), and sensorimotor cortex (SM). In the sham group, all four clusters were localized and bilaterally symmetric and were consistent across all time points studied. In the TBI group, with the exception of the Cg cluster, the clusters were comparatively less localized and showed a mixture of uni- and bilateral rsfMRI patterns. Some clusters showed partial recovery with time, but others did not. Note that in the TBI group, two separate but related components were observed as shown in panels (B) and (**C**). ICA, independent component analysis; TBI, traumatic brain injury.

Figure 2 compares the rsfMRI Z-score maps of the four ICA components between sham and TBI animals. In the sham group, the Cg, CPu, and SSS clusters were bilaterally symmetric and were consistent across all time points studied. The SM cluster showed slightly less symmetric rsfMRI patterns on days 2, 7, and 14 compared to other ICA clusters, suggesting that the sham surgical operation might have slightly altered its functional connectivity in this region.

In the TBI group, the Cg cluster was similar to the sham group across all time points studied, suggesting that the Cg was not affected in this injury model. The CPu cluster appeared relatively diffuse and less symmetric compared to the sham group at all time points. The SSS cluster showed a diffuse bilateral pattern on day 0 (3 h post-TBI), two unilateral patterns in the opposite hemisphere (Fig. 2B,C) on day 2, and returned to toward normal bilateral patterns on day 14 (Fig. 2C), suggesting that there was some initial IFC disruption and eventually complete recovery with time. The SM cluster (closest to the impacted area) had its own coherence and was predominantly unilateral on day 0 and subsequent time points (Fig. 2B), suggesting abnormal IFC. There was also a corresponding unilateral cluster in the contralesional hemisphere at all time points (Fig. 2C). The bilateral patterns were absent on days 0 and 2, but returned on days 7 and 14 (Fig. 2C), suggesting partial recovery with time. Overrall, the SM cluster showed more severe IFC disruption and less recovery compared to the SSS cluster.

### Interhemispheric connectivity

**FIG. 3.**
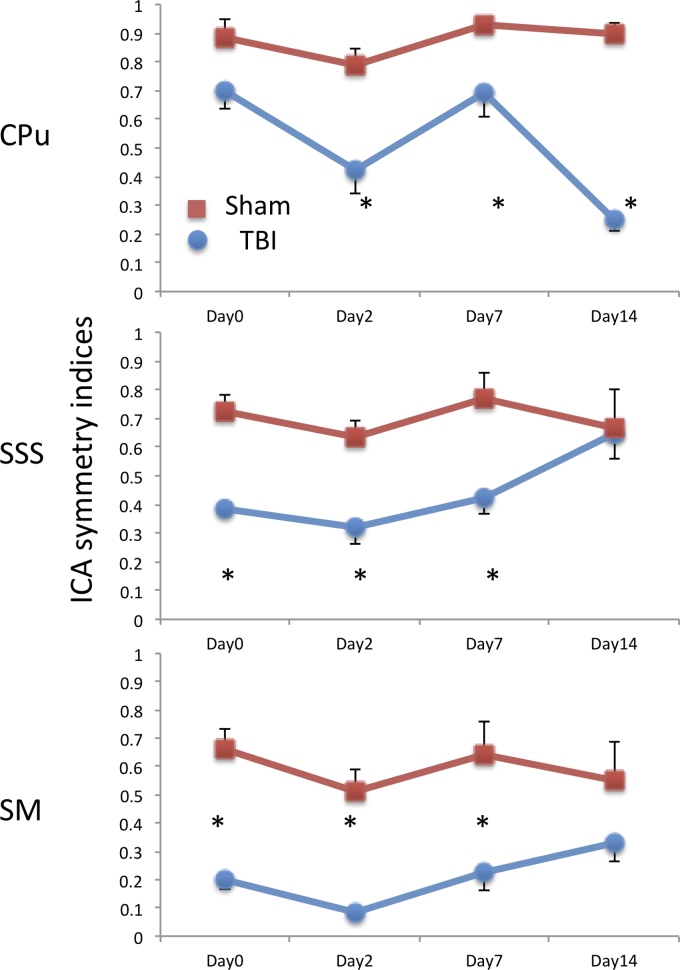
ICA symmetry index. Volume ratios of the contra- to ipsilesional ICA clusters were tabulated. Unity indicates symmetry. In the sham group, the ICA symmetry index across all time points and clusters was close to unity. In the TBI group, the ICA symmetry indices varied substantially across time. Data are expressed as mean ± SEM, *n* = 9. *Indicates *p* < 0.05 from sham. Red denotes shams, and blue denotes TBI animals. ICA, independent component analysis; SEM, standard error of the mean; TBI, traumatic brain injury.

To quantify the IFC, interhemispheric symmetry indices (SI, defined as the hemispheric volume ratios of the rsfMRI clusters) were analyzed for the four ICA clusters (Fig. 3). In the sham group, the Cg SI was not statistically different from unity and was time invariant as expected (data not shown). The CPu SI was not statistically different from unity and was time invariant. The SSS and SM SIs were slightly below unity, suggesting that the sham surgical operation might have slightly affected its functional connectivity in this region.

In the TBI group, Cg SI was not statistically different from unity and was time invariant (*p* > 0.05, data not shown), indicating that this brain structure was not significantly affected in this TBI model. Mixed-effects model analysis of comparison between the TBI and sham group shows that the *p* values for group factor, time point factor, and group*time point factor for CPu were 0.000, 0.001, and 0.004; for SSS they were 0.000, 0.105, and 0.082; and for SM they were 0.000, 0.207, and 0.433. These results indicated that TBI group SIs at three locations were all significantly different from the sham group. The CPu also showed a significant difference over time and significant interaction, indicating that the temporal changes were different between TBI and sham groups, whereas there were not significant effects attributable to time detected in the SSS and SM. To find out the statistical differences at different time points, the *t*-test with multi-comparison correction was performed.

The CPu SI was relatively normal on day 0 (*p* > 0.05), but became abnormal by day 14 (*p* < 0.05), suggesting some deterioration in IFC in the CPu. The SSS SI was significantly different on day 0 (*p* < 0.05), worsened on day 2 (*p* < 0.05), but was not significantly different from sham on day 14 (*p* > 0.05). The SM SI was significantly smaller than sham on day 0 (*p* < 0.05), worsened on day 2 (*p* < 0.05), and recovered somewhat with time, but remained below (albeit not significantly) the sham values on day 14.

Together, these results suggest that there were widespread disruptions in IFC networks in areas close to and distant from the impact site. There was also evidence of partial recovery of some of the functional networks.

### T_2_ and apparent diffusion coefficient

**FIG. 4.**
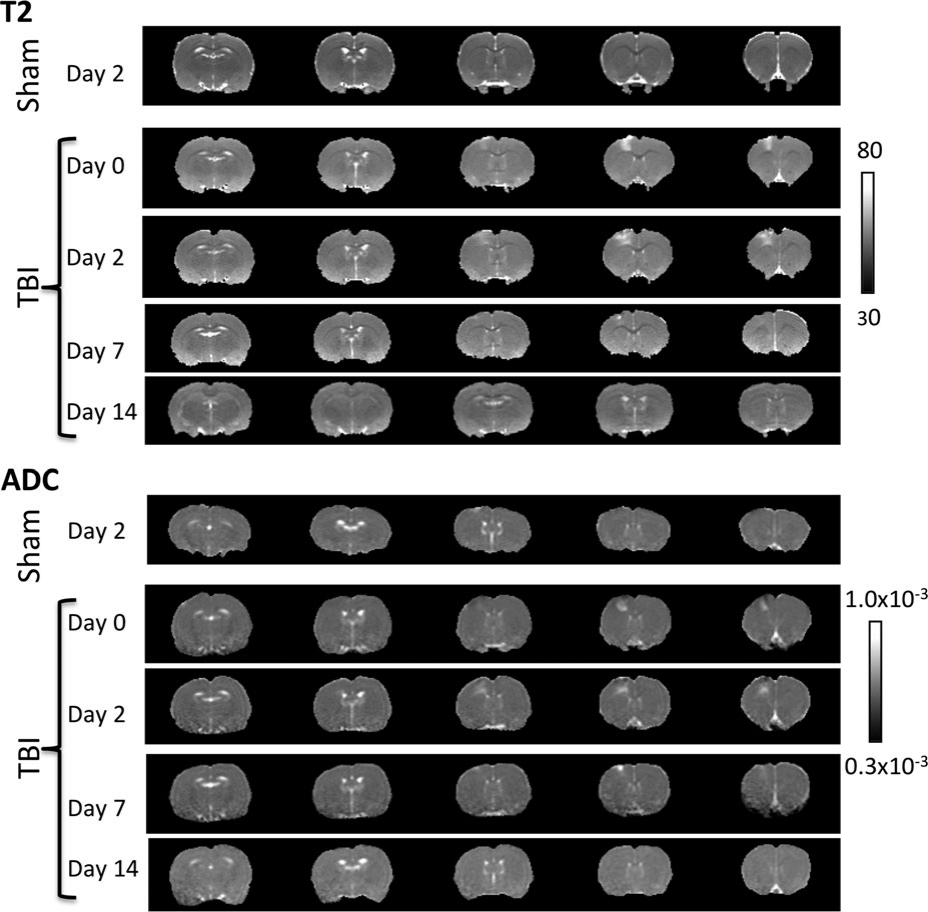
T_2_ and ADC maps of a representative sham animal on day 2 and a TBI animal at multiple time points. Sham animals showed no apparent lesion on day 2, except the very superficial cortical surface. The TBI animal showed abnormal and evolving T_2_ and ADC in the impact area, peaking on day 2, and subsided on day 7 and largely disappeared by day 14. ADC, apparent diffusion coefficient; TBI, traumatic brain injury.

To determine whether the abnormal rsfMRI patterns had underlying structural changes, T_2_ and ADC of the ICA clusters were analyzed. Figure 4 shows the T_2_ and ADC maps of a representative sham animal on day 2 and a TBI animal at multiple time points. Sham animals showed no T_2_ or ADC abnormalities at any time point, except some ADC hyperintensity at the very superficial cortical surface in certain animals at some time points. By contrast, the TBI animals showed abnormal T_2_ and ADC deep in the cortex that evolved spatially and temporally.

In the contralesional hemisphere, quantitative analysis showed that group T_2_ and ADC were not significantly different across time, were within normal range, and did not differ from sham (*p* > 0.05, data not shown). The mean T_2_ and ADC of the contralesional hemisphere were 56.5 ± 1.9 ms and (0.735 ± 0.047) × 10^–3^ mm^2^/s, respectively (average across all time points and sham animals). TBI ipsilesional ROIs were thus normalized with respect to homologous regions in the contralesional hemisphere to simplify comparisons.

**FIG. 5.**
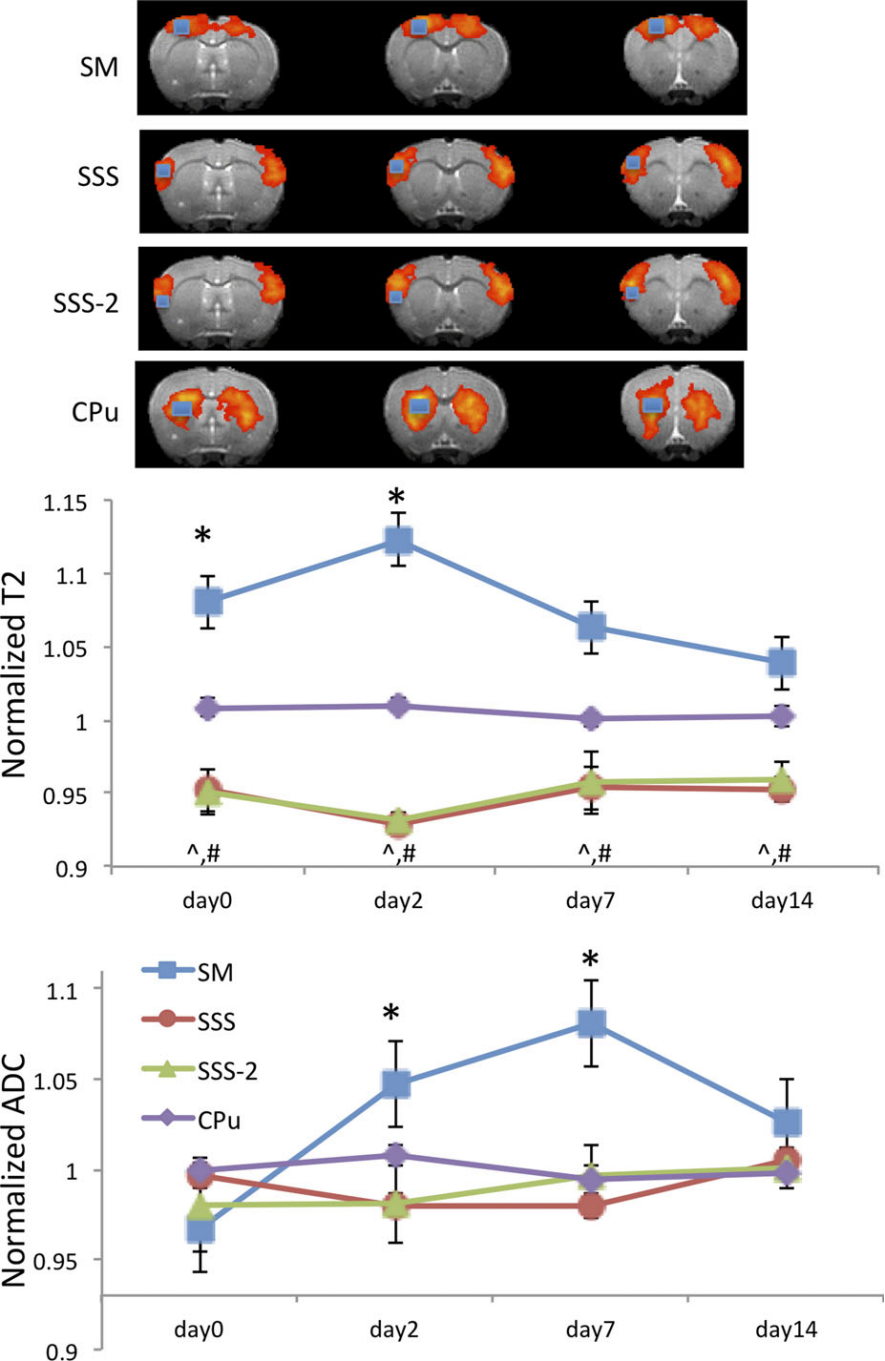
Normalized T_2_ and ADC of the supplementary somatosensory cortex (SSS), sensorimotor cortex (SM), an ROI next to the SSS (SSS-2), and the CPu on days 0 (3 h), 2, 7, and 14 after TBI (mean ± SEM, *n* = 6). T_2_ and ADC values were tabulated for the four ROIs based on the rsfMRI maps Cg, SM, SSS, and CPu. ^*,^,#^Indicate significant differences (*p* < 0.05) from contralesional values. ADC, apparent diffusion coefficient; Cg, cingulate; CPu, caudate putamen; ROI, region of interest; rsfMRI, resting-state functional magnetic resonance imaging; SEM, standard error of the mean; TBI, traumatic brain injury.

Normalized group T_2_ and ADC of ICA clusters in the TBI group are plotted in Figure 5. Normalized T_2_ of the SM ROI was elevated on day 0 (*p* < 0.05 from the contralesional value), peaked on day 2 (*p* < 0.05), and returned toward normal value on day 14 (*p* > 0.05), indicative of evolving vasogenic edema. Normalized T_2_ of the SSS ROI was, surprisingly, 5% lower than contralesional values (*p* < 0.05) at all time points (possibly attributable to edema in the impact site pressing against this neighboring tissue). To corroborate, two additional ROIs were analyzed. Normalized T_2_ from an ROI next to the SSS, SSS-2 also showed a similar pattern (*p* < 0.05), whereas that of the CPu ROI, which was farther away from the impact site, did not (*p* > 0.05). The largest differences between SSS and SSS-2 were found on day 2, consistent with the peak of vasogenic edema.

The SM ROI showed patterns of reduced ADC on day 0 (*p* < 0.05), elevated ADC on days 2 and 7 (*p* < 0.05), and returned toward normal on day 14 (*p* > 0.05). The SSS and SSS-2 ADC values were slightly below, but not significanlty different from unity (*p* > 0.05). CPu ADC values were not significantly different from normal (*p* > 0.05) and time invariant (*p* > 0.05).

T_2_ and ADC values for the Cg cluster in the TBI group were “normal” as expected and time invariant (data not shown). They were not analyzed further.

### Lesion volume

**FIG. 6.**
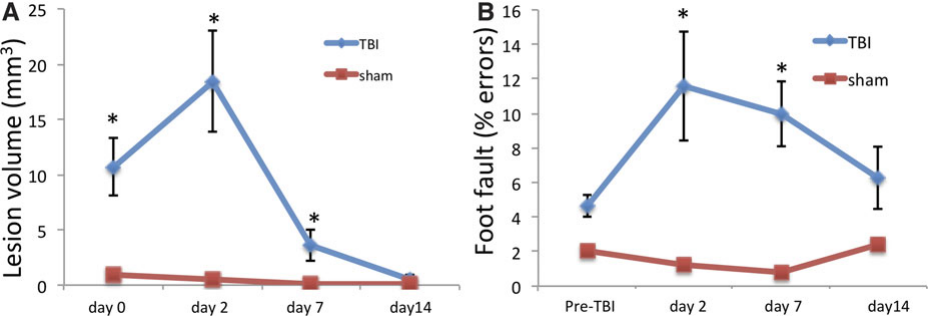
(**A**) T_2_ lesion volume of sham (*n* = 6) and TBI (*n* = 9) animals at days 0, 2, 7, and 14 days after TBI. Data are expressed as mean ± SEM. *Indicates *p* < 0.05 from the sham group. (**B**) Foot-fault scores for subjects at pre-TBI and 2, 7, and 14 days post-TBI. Data are expressed as mean ± SEM. *Indicates *p* < 0.05 from the sham group. SEM, standard error of the mean; TBI, traumatic brain injury.

Group T_2_ lesion volume in the TBI group was apparent on day 0 (*p* < 0.05), peaked on day 2 (*p* < 0.05), and remained toward normal by 14 days (*p* > 0.05) after TBI (Fig. 6A). By comparison, the sham group showed no apparent lesion on T_2_ and ADC.

### Behavioral scores

For the TBI group, behavioral scores were significantly different from those of the sham group on days 2 and 7 (*p* < 0.05), but not on day 14 (*p* > 0.05; Fig. 6B). By comparison, for the sham group, foot-fault behavioral scores showed no significant differences among pre-TBI and post-TBI at all time points (*p* > 0.05).

### Correlation analysis

The rsfMRI SIs were correlated with behavioral scores, lesion volume, T_2_ values, and ADC values across time for the SM, SSS, and CPu ROIs in the TBI group (Table 1). For the SM cluster, the rsfMRI asymmetry index was significantly correlated with behavioral score (*p* < 0.05), lesion volume (*p* < 0.05), and T_2_ (*p* < 0.05), but not ADC (*p* = 0.09). For the SSS cluster, the rsfMRI asymmetry index was significantly correlated with behavioral score (*p* < 0.05), but not lesion volume (*p* > 0.05), T_2_ (*p* > 0.05), or ADC (*p* > 0.05).

**Table 1.** Pearson Correlation of rsfMRI Interhemispheric Connectivity Index with Lesion Volume and Foot-Fault Score in the CPu, SSS, and SM

	CPu		SSS		SM	
	*r*	*p*	*r*	*p*	*r*	*p*
Lesion volume	0.1373	0.8627	–0.8509	0.1491	–0.9831	0.0169
Foot fault	–0.5361	0.4639	–0.9932	0.0068	–0.9304	0.0500
T_2_	na	na	0.5382	0.4618	–0.9999	0.0007
ADC	na	na	0.8104	0.3985	–0.9896	0.0920

Correlation of the rsfMRI data with T_2_ and ADC of the CPu was not performed because no changes in T_2_ and ADC were observed. *r* is the Pearson correlation coefficient with the corresponding two-tail *p* value. “na” indicates not analyzed given that no significant correlation was expected.

rsfMRI, resting-state functional magnetic resonance imaging; CPu, caudate putamen; SSS, supplementary somatosensory cortex; SM, sensorimotor cortex; ADC, apparent diffusion coefficient.

## Discussion

This study investigated longitudinal changes in rsfMRI IFC after a moderate TBI of the unilateral SM in rats using ICA. In a moderate TBI model, the changes in brain function last longer than a few minutes post-trauma. Symptoms may be similar to a mild TBI, but the symptoms do not go away or may even get worse. We focused on interhemispheric connectivity because that showed the strongest change in this TBI model. The major findings are: 1) TBI animals showed less localized rsfMRI patterns in the sensorimotor network compared to sham and control animals; 2) ICA rsfMRI clusters in the sensorimotor network showed less bilateral symmetry compared to sham and normal animals, indicative of IFC disruption; 3) with time after injury, some of the rsfMRI patterns in the sensorimotor network showed more bilateral symmetry, indicative of improvement in IFC with time; and 4) in the SM, IFCs were correlated with changes in behavioral scores, lesion volume, and T_2_, but not diffusion. In the supplementary sensory cortex, IFCs were only correlated with changes in behavioral scores. In sum, rsfMRI revealed widespread disruption and partial recovery of the IFC in the sensorimotor network post-TBI, and these changes correlated with behavioral deficit and recovery, but less so to structural changes.

### Clinical relevance of the traumatic brain injury model

The controlled cortical impact injury (CCI) model of TBI was chosen for this study because it is more accurate, easier to control, and produces brain injuries similar to those observed in humans compared to other TBI models (weight drop and fluid percussion injury). Further, based on the impact parameters, varying severity levels of injury can be achieved in a more consistent manner, thereby improving the power of the study. The CCI model has been widely utilized to study TBI in a variety of ways, including: 1) to evaluate the potential of novel therapeutic compounds and therapeutic window of administration; 2) to define pathophysiological changes in association with TBI; 3) to study TBI across a life span (from immature to aged animals); 4) to determine the functional outcomes associated with TBI; and 5) the consequence of repetitive TBI, among others. The moderate TBI is also commonly used in humans, and, previously, human studies of moderate TBI have found axonal injury, disrupted blood–brain barrier, edema formation, alterations in blood flow, histological changes, inflammatory response, and functional outcomes.^29–31^ Therefore, this CCI is an appropriate model to further study the effect of TBI on alterations in rsfMRI.

### Sham and control group

In the sham group, the rsfMRI patterns in the Cg, SSS, and CPu were highly localized, bilateral and stable across time, lending confidence to the rsfMRI protocol and the analysis approach. The SM rsfMRI cluster, however, is slightly asymmetric, likely related to the sham operation. This is confirmed by data from a separate group of normal animals without surgery, in which all four clusters were consistently bilaterally symmetric.

### Traumatic brain injury group

rsfMRI patterns of the Cg were not affected in this TBI model, serving as an internal control brain structure for rsfMRI analysis. By contrast, rsfMRI patterns of the CPu, SSS, and SM in the TBI group on day 0 were diffuse and asymmetric compared to those of the sham group across all time points. SM IFC was most disrupted as expected because the SM cluster was located under the impact site. There were T_2_ and ADC abnormalities in this region that peaked on day 2. We have previously reported similar T_2_ and ADC abnormality profiles,^16^ marked hypoperfusion on day 0, hyperperfusion on day 2, and mild hypoperfusion at 7 and 14 days^32^ in a similar brain region. Note that our T_2_ and ADC changes were smaller than those reported previously,^16^ which is expected because the ROIs differed (ours were based on ICA cluster, and Long and colleagues' were based on T_2_ hyperintensity). Nonetheless, these structural and hemodynamic abnormalities support the severe IFC disruption in the SM cortex.

SSS IFC was disrupted despite it not being located under the impact area, suggesting a functional connection between the SSS cluster and the impact site or indirect effects of the impact. SSS IFC returned toward sham values on day 14, suggesting recovery with time. There were no abnormal T_2_ or ADC in this region. We previously found no T2 or apparent diffusion abnormalities,^16^ but there was mild hypoperfusion on days 0–7 (∼75–80% of normal) that normalized by 14 days^32^ in a similar brain region.

CPu IFC was normal on day 0, but became abnormal on day 14, suggesting deterioration with time. We were surprised by the extent of abnormal SI in the CPu. There were no gross T_2_ or ADC abnormalities at any time point studied. A previous study found no abnormal T_2_ and ADC,^16^ but did find mildly abnormal cerebral blood flow^16^ in the CPu. The CPu is further away from the impact site, but it is a part of the sensorimotor network (functionally and structurally connected to the SM). Thus, it is conceivable that its IFC was disrupted. CPu IFC disruption is consistent with mild behavioral deficits that persisted on day 14, although this is not the only possible contribution to the residual functional deficits.

Altogether, these observations indicate that TBI markedly disrupts the IFC in the SM and mildly disrupts the IFC in the SSS and CPu. There is partial recovery in the SM and almost complete recovery in the SSS. The mechanism(s) of such recoveries could be attributable to functional reorganization and/or functional compensation. This could arise from shifts or changes in neural activity in the ipsi- and contralesional cortices as a result of the strengthening of existing pathways, forming of new pathways, or the rearranging of pathways in adjacent and distant regions to the injured site after brain injury.^33,34^ It could also be attributable to recovery of temporary interruption through direct callosal connections, such as transhemispheric diaschisis.^35^

### Edema effects

We were surprised by the small but significant T_2_ reduction (5%) in the SSS ROI. This is likely attributable to edema from the TBI pressing against neighboring tissues and reducing extracellular water and thus T_2_. To corroborate, two additional ROIs were analyzed. The T_2_ of an ROI next to the SSS, SSS-2, showed a similar pattern, but that of the CPu, which is farther away from the impact, was normal. Similarly, the ADC in the SSS and SSS-2 were also slightly, albeit not significantly, reduced compared to contralesional values, possibly because the ADC had less sensitivity than T_2_. The ADC of CPu was normal, also consistent with T_2_ finding.

### Correlation analysis

In the SM, changes in IFC were correlated with behavioral scores, lesion volume, and T_2_, but not ADC. The lack of significant correlation with ADC (*p* = 0.09) may be attributable to the biphasic pattern of ADC, namely, that ADC is reduced on day 0 relative to control but increased on day 2. This profile change is more dramatic than that of the SI, thereby weakening the correlation. Mismatch of anatomical and functional pixels could also weaken the correlation. In the SSS, IFC changes were only correlated with changes in behavioral scores, but not with lesion volume, ADC, and T_2_, which is consistent with the SSS showing no apparent structural abnormality.

Although the temporal profiles are similar, the behavioral scores do not completely recover on day 14 whereas lesion volumes mostly disappear (residual damage and cell death might persist). This apparent difference is not unexpected because they measure different aspects of the injury. Previously studies have reported differences in outcomes between function and infarct volume in ischemic stroke.^36–38^

### Other studies

Although many human rsfMRI TBI studies have investigated changes in various brain regions (see for review a previous work^3^), only a few studies have reported abnormal IFC with rsfMRI.^39,40^ After a single concussive blow, subjects showed significantly reduced IFC in the primary visual cortex and hippocampal and dorsolateral pre-frontal cortex networks.^39^ In another study, in patients with consistent traumatic axonal injury and acute subcortical white matter abnormalities, rsfMRI found significantly lower IFC for the hippocampus and anterior cingulate cortex.^40^ Many human TBI rsfMRI studies report changes in the DMN. Although the DMN in rats has been reported on using very light anesthesia,^41^ we did not consistently detect DMN patterns in this study.

By contrast, there have been very few rsfMRI studies done on animal TBI models. In a rat TBI model,^13^ rsfMRI analysis with seed ROIs in the infralimbic cortex, and in the anterior cingulate cortex, found diminished connectivity between cortical areas involved in cognition. The connectivity patterns normalized at 1 and 3 weeks post-injury. Correlation coefficients were used to evaluate connectivity strength. The Morris water maze test showed that cognitive deficits persisted for the first week after injury and largely resolved thereafter. In another animal study,^12^ TBI was induced by lateral fluid percussion injury, and rsfMRI using seed-based ROIs found decreased connectivity between the ipsi- and contralateral parietal cortex as well as between the parietal cortex and hippocampus on the side of injury as compared to sham-operated animals. Injured animals also had abnormal negative connectivity between the ipsi- and contralateral parietal cortex and other regions. The pentylenetetrazol test revealed increased seizure susceptibility in TBI compared to sham rats. Harris and colleagues^15^ acquired rsfMRI data at days 7, 14, and 21 in a unilateral CCI adult rat model of TBI. A two-tailed statistical analysis over multiple brain regions in a data-driven approach was used to assess global and regional changes in network topology. Besides the somewhat anticipated reductions in functional connectivity within the interhemispheric and corticothalamic circuitry, they also reported the unanticipated finding of regional hyperconnectivity, which persisted chronically in some sensorimotor and subcortically connected regions. Harris and colleagues did not investigate acute and subacute phases of TBI. In another study,^14^ a closed-head momentum exchange model was used to produce one or three mild TBIs in young adult male rats. The rsfMRI data were also analyzed using a seed-based method. They found that disruption of functional connectivity was pronounced with three mild hits. Interestingly, rats exposed to one hit showed *enhanced* functional connectivity (or hyperconnectivity) across brain sites, particularly between the olfactory system and cerebellum. Our study reported the first disruption and subsequent recovery in the IFC of the sensorimotor pathway in rats, along with correlations with behavioral scores and other MRI measures. IFC disruptions have been reported in experimental stroke.^42,43^

### Limitations

There are several limitations of this study: 1) We only focused on analyzing the sensorimotor networks in this TBI model because they are relevant to the impact location and are robust based on the control animals. Other networks could also be affected by TBI. 2) Whereas the interhemispheric volume ratio of the ICA clusters was used, the Z-score ratio should yield similar conclusions. 3) The SIs were taken as the smaller number over the larger number of the two hemisphere. Although this choice could create a small offset from unity, such an offset is not a significant issue because comparisons were made with the sham group. 4) Whereas seed-based analysis offers different advantages, we found the rsfMRI results to be highly dependent on ROI placement (data not shown). 5) We investigated only the open skull TBI model as a first step; future studies will investigate a repeated closed-skull TBI model as well as chronic effects of TBI.

## Conclusion

This study investigated rsfMRI functional connectivity after a moderate controlled impact to the unilateral SM in rats. rsfMRI revealed apparent and widespread disruption in the interhemispheric functional network connectivity of the sensorimotor pathway and its subsequent recovery. These changes correlated with behavioral changes, but less so to structural changes. IFC may prove useful for informing functional brain reorganization that may attribute to behavioral changes, as well as for monitoring novel therapeutic interventions.

## Abbreviations Used

ADC: apparent diffusion coefficient
BOLD: blood oxygenation level–dependent
CCI: controlled cortical impact injury
Cg: cingulate
Cpu: caudate putamen
DMN: default mode network
EPI: echo-planar imaging
fMRI: functional magnetic resonance imaging
FOV: field of view
ICA: independent component analysis
IFC: interhemispheric functional connectivity
MRI: magnetic resonance imaging
ROIs: regions of interest
rsfMRI: resting-state functional magnetic resonance imaging
SI: symmetry index
SM: sensorimotor cortex
SPM: Statistical Parametric Mapping
SSS: supplementary somatosensory cortex
T: Tesla
TE: echo time
TR: repetition time
